# Geospatial analysis and participant characteristics associated with colorectal cancer screening participation in Alberta, Canada: a population-based cross-sectional study

**DOI:** 10.1186/s12913-023-10486-8

**Published:** 2023-12-21

**Authors:** Geneviève Jessiman-Perreault, Jessica Law, Kamala Adhikari, Amanda Alberga Machado, Barbara Moysey, Linan Xu, Huiming Yang, Lisa K. Allen Scott, Gary Teare, Alvin Li

**Affiliations:** 1 Provincial Population and Public Health, Alberta Health Services, Holy Cross Centre, 2210 2 St SW, Calgary, AB T2S 3C3 Canada; 2 Department of Community Health Sciences, University of Calgary, 3280 Hospital Drive NW, Calgary, AB T2N 4Z6 Canada; 3 Screening Programs, Provincial Population and Public Health, Alberta Health Services, Holy Cross Centre, 2210 2 St SW, Calgary, AB T2S 3C3 Canada; 4 Department of Oncology, University of Calgary, 1331 29th Street NW, Calgary, AB T2N 4N2 Canada

**Keywords:** Colorectal cancer, Screening, Prevention, Health system, Geographic, Hotspots

## Abstract

**Background:**

Colorectal cancer (CRC) is a leading cause of death in Canada and early detection can prevent deaths through screening. However, CRC screening in Alberta, Canada remains suboptimal and varies by sociodemographic and health system characteristics, as well as geographic location. This study aimed to further the understanding of these participant and health system characteristics associated with CRC screening in Alberta and identify clusters of regions with higher rates of overdue or unscreened individuals.

**Methods:**

We included Albertans aged 52 to 74 as of December 31, 2019 (index date) and we used data from administrative health data sources and linked to the Alberta Colorectal Cancer Screening Program database to determine colorectal cancer screening rates. We used multivariable multinomial logistic regression analysis to investigate the relationship between sociodemographic, health system characteristics and participation in CRC screening. We used optimized Getis-Ord Gi* hot-spot analysis to identify hot and cold-spots in overdue for and no record of CRC screening.

**Results:**

We included 919,939 Albertans, of which 65% were currently up to date on their CRC screening, 21% were overdue, and 14% had no record of CRC screening. Compared to Albertans who were currently up to date, those who were in older age groups, those without a usual provider of care, those who were health system non-users, and those living in more deprived areas were more likely to have no record of screening. Areas with high number of Albertans with no record of screening were concentrated in the North and Central zones.

**Conclusions:**

Our study showed important variation in colorectal cancer screening participation across sociodemographic, health system and geographical characteristics and identified areas with higher proportions of individuals who have no record of screening or are under-screened in Alberta, Canada.

**Supplementary Information:**

The online version contains supplementary material available at 10.1186/s12913-023-10486-8.

## Background

Colorectal cancer (CRC) is the second most common cancer and a leading cause of death from cancer in Canada [1]. Early detection can prevent deaths from colorectal cancer by using screening tools such as fecal immunochemical tests (FIT) and colonoscopies [2–3]. In Alberta, Canada, FIT is recommended for CRC screening every 1–2 years for average risk asymptomatic individuals aged 50–74 years old, and every 10 years for people with increased for CRC (i.e., individuals who have a family history of CRC or personal history of CRC and/or adenomas or have inflammatory bowel diseases) [4]. If an individual receives a positive FIT test, they are also referred for colonoscopy [5]. FIT has been used as a CRC screening tool in Alberta since November 2013 and has been offered to all average risk individuals aged 50–74 [5]. However, the uptake of FIT remains suboptimal [6–7] and uptake varies considerably by sociodemographic factors such as individual ethnicity, educational attainment, language spoken, income, and marital status [8–11]. Moreover, health system access factors such as having a regular general practitioner (GP) and frequency of GP visits have been found to be associated with higher rates of CRC screening [7, 12–13].

Geography is another common barrier in accessing cancer preventative services. Individuals residing in rural communities are less likely to have a regular general practitioner [14], make fewer visits to general practitioners [15], and travel further distances to seek care [16]. Therefore, longer distances to healthcare services may be an important barrier to CRC screening, particularly in a country such as Canada, which has a relatively small population spread across a large land mass. Kurani, McCoy and Kampman [17] focused on the geographic variation of CRC screening, and its association with other socioeconomic factors in other jurisdictions such as in the American Midwest and found that individuals living in more rural areas with more deprivation had lower screening rates but similar analyses of geographic and socioeconomic factors’ role in CRC screening participation have not been examined in Canada.

Within Canada, socioeconomic, health system and geographic predictors of CRC screening have been examined separately, but few studies, and none in Alberta, have examined these factors together to see how they contribute to CRC screening patterns. Moreover, this information will be used within the provincial healthcare authority to inform the design and implementation of interventions aimed at increasing uptake of CRC screening among subpopulations with low screening. The aim of this study is to understand the individual and geographic factors associated with CRC screening in Alberta, Canada, using administrative data. To fulfil this aim, we first examined the association of different socioeconomic and health system factors with CRC screening. Second, we identified clusters of regions with higher rates of individuals who were overdue for CRC screening or had no record of CRC screening (i.e., hot spots).

## Methods

### Study design overview

This is a population-based cross-sectional study, that used multiple administrative health data sources and linked to the Alberta Colorectal Cancer Screening Program (ACRCSP) database. The total study population was 919,939.

### Data source and data linkage

To create the dataset for this study we linked multiple datasets from Alberta Health Services (AHS) using the individuals’ Personal Healthcare Number (PHN) or Unique Lifetime Identifier (ULI). Information on the screening status was taken from the ACRCSP data, collected by AHS screening program. The database includes key information on colonoscopy status, fecal immunochemical test (FIT) including date of testing, whether patients were mailed a screening letter to inform them to get tested and results of the FIT test. These data were linked with three administrative sources of health care utilization data: (1) the National Ambulatory Care Reporting System (NACRS), (2) the Physicians Claims dataset, and (3) the Discharge Abstract Database (DAD). We included data from 1 year prior to the index date (i.e., December 31, 2019) to define comorbidity and 5 years prior to the index date to define healthcare utilization. The NACRS contains health administrative, demographic, clinical and service specific data for all hospital- and community-based ambulatory care including day surgery, emergency department, outpatient, specialty care, and community clinic visits. This data source was linked to CRC screening status to ascertain healthcare utilization data (e.g., mental health services, comorbidity). The Physicians Claims dataset contains the date of service and related diagnostic and treatment information submitted for fee-for-service billing. Physician specialty information is available in these databases. The Discharge Abstract Database (DAD) contains a record for every inpatient hospitalization in the province. The DAD contains health administrative, demographic, and clinical data including discharges, deaths, and transfers from acute care institutions. Finally, the Alberta Health Care Insurance Plan (AHCIP) Provincial Registry contains population demographics for all persons covered for basic medical and hospital insurance during a given fiscal year. This data source was used as a proxy denominator for populations under study.

To obtain data to generate geographic and access to health services variables, we used a Network Dataset comprised of Alberta Health Postal Code Translator File, DMTI Route Logistics Road New File, Alberta Municipality Data Sharing Partnership (AMDSP) road data from 2013 and AHS Facility Locations. Finally, the Pampalon Deprivation index, derived from the Canadian census data, is a small area based composite measure of socioeconomic status aggregated at the dissemination area (DA) level [18].

### Inclusion and exclusion criteria

**Fig. 1:**
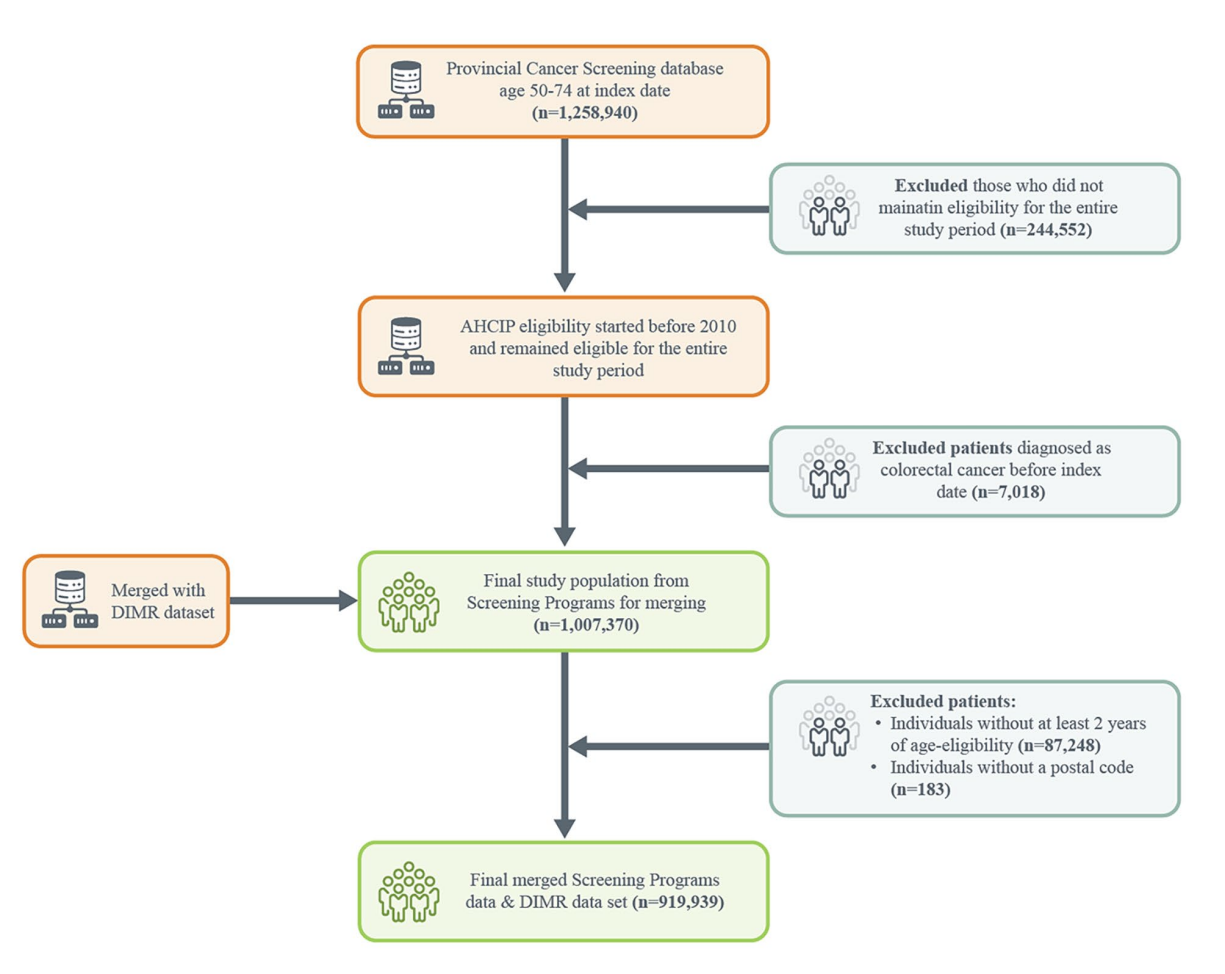
Flowchart of Study Dataset Creation *(*Alberta Health Care Insurance Plan (AHCIP); Data Integration, Measurement & Reporting (DIMR))

Once the linked administrative dataset was generated, we applied the following inclusion and exclusion criteria: first, we included Albertans aged 52 to 74 as of December 31, 2019 (i.e., the index date). While the guidelines recommend screening for those 50 to 74, we restricted our sample to individuals at least aged 52 to ensure individuals had at least two years in the age-eligible screening window (n = 87,248). Second, we excluded those who had been diagnosed with colorectal cancer prior to the index date (n = 7,018). Third, we only kept records for Albertans who had continuous registration with the province for 10 years (since January 1, 2010) to capture an accurate screening status by excluding those who may have screened elsewhere prior to immigrating to the province. We also excluded any individuals for whom postal code was not found in the data (n = 183). The final dataset included data from 919,939 individuals (see Fig. 1 for information on the dataset creation).

### Covariates

We grouped our covariates into three categories of factors: (1) demographic and socioeconomic factors (i.e., age, sex, Pampalon social and material deprivation), (2) patient health and health system factors (number of visits to GP, continuity of care, auto travel distance to closest lab in minutes, health profile group), and (3) geographic factors (zone, local geographic area (LGA), rural/urban regions).

### Demographic and socioeconomic variables

We included patient data on age in years (as of the index date) and sex (male, female). Socioeconomic status was measured by using the two subcomponents of the Pampalon Deprivation index. The Pampalon Deprivation index is made of a social component and a material component classifying the population’s area of residence into quintiles according to a level of deprivation [17]. The Pampalon material deprivation index is a composite measure of small area SES that combines the proportion of persons without high school diplomas, the average personal income, and the rate of unemployment within the DA. The social component reflects the deprivation of relationships among individuals in the family, and the community and the material component illustrate deprivation of wealth, goods, and convenience.

### Patient health and health system factors

Usual provider of care (UPC) is a measure used in the study to assess continuity of care over a period of time, this variable was generated based on data extracted from the Physician Claims dataset. A UPC continuity score was calculated for individuals who had at least three visits to a family or primary care physician within the 3 years prior to study index date This score was used to create four levels of UPC (no UPC, low UPC, moderate UPC, and high UPC) among those who had a usual provider of care [19]. The physician with the most visits was considered as the regular general practitioner (GP) and the continuity score is calculated by using the number of visits with the regular physician in the 3 years prior to the index date and dividing by the total number of visits with any community GP in the same period. Patients with less than 3 visits to their regular physician was classified as having no UPC. Those classified at low UPC have a continuity score between 0 and 0.4, those with moderate UPC have a score between 0.4 and 0.7 and high is UPC is between 0.7 and 1. Multiple billing records on the same day were counted as a single visit.

Driving distance to nearest health services laboratory was calculated using the comprehensive Alberta Facilities Distance/Time Look Up Version 3 2020 table created by AHS. This look-up table was used to calculate the driving distance of the individual’s postal code address to the closest health services laboratory. These distances were then categorized into four groups (Between 0 and 10 min, 10 + to 20 min, 20 + to 30 min, > 30 min). We chose distance to health services laboratory because in Alberta, Canada, FIT tests are mostly picked up and/or drop off at these locations.

The Canadian Institutes for Health Information (CIHI) Population Grouper Methodology summarizes type of health care utilization by placing individuals into health risk groups based on history of health services [20]. This variable is generated using administrative data records from inpatient, emergency department ambulatory, and physician claims. The health risk groups provide broad information about the type (e.g., acute, chronic, cancer) and severity (e.g., moderate, major) of the health profile group.

### Geographic variables

Using the patient postal code, each person was assigned to an AHS Zone (North, Edmonton, Central, Calgary, South) and a LGA using the Alberta Health Postal Code Translator File. In Alberta there are 132 LGAs of varying size divided across in fives health zones (North Zone, Edmonton Zone, Central Zone, Calgary Zone, and South Zone). Rural/urban regions is 7-category geography variable generated by combining LGAs based on criteria such as population density, distance from urban or rural centres that provide health and non-health services, travel patterns of populations who are seeking health care, and commuting behaviours [21]. The seven categories consist of: (1) metro centres (i.e., population of above 500,000), (2) metro influenced (i.e., commuter communities surrounding metro centres), (3) urban (i.e., urban centres with populations of more than 25,000 but less than 500,000), (4) moderate urban influenced (i.e., areas surrounding the urban centres), (5) rural centre areas (i.e., areas with a population of more than 10,000 but less than 25,000), (6) rural (i.e., areas with populations with less than 10,000 and up to 200 kms form urban or metro areas), and (7) rural remote (i.e., greater than 200 kms from urban or metro centres) [21].

### Outcome variables

Our primary outcome variable was CRC screening status. We classified individuals into three categories of CRC screening: (1) Currently up-to-date (CUTD) (i.e., the individual had at least one FIT test within the last two years of index date or the individual had a colonoscopy within the last 10 years of the index date); (2) No record of screening (i.e., if the individual had no record of a FIT test or colonoscopy ever in Alberta); (3) Overdue (i.e., individuals not in either CUTD or the no record of screening category are classified as overdue).

### Statistical analysis

First, we examined characteristics of the study population using descriptive statistical analysis (i.e., frequencies, percentages, medians, and interquartile range) on study variable by the three levels of CRC screening (CUTD, No record, Overdue). Next, we used crude multinominal logistic regression (data not shown) followed by an adjusted multinomial logistic regression model, including simultaneous adjustment for all study covariates to investigate the demographic and socioeconomic, patient health and health system, and geographic factors associated with having no record of screening and being overdue for screening using CUTD as a referent group. Exposure factors were determined a priori based on their association with cancer screening in past literature [7–16]. We considered statistical significance based on an alpha level of 0.05. All statistical analysis was conducted using R version 4.0.2.

### Spatial analysis

Individual postal code was geocoded using the Alberta Health Postal Code Translator File and estimates for rate of overdue for and no record of CRC screening were aggregated at the Local Geographic Area (LGA). The LGA was chosen for this spatial analysis, as they are the lowest geographic area that AHS and Alberta Health use as official geographies [21]. These LGAs provide sufficient sample size to allow for accurate and precise estimates and have been used in other geospatial studies of health outcomes in Alberta [22–24]. Using these aggregate estimates of overdue for and no record of CRC screening, choropleth maps were generated using Tableau Desktop Version 2021.2.1.

Next, Global Moran’s I was used to detect whether spatial autocorrelation exist. Essentially, this metric is used to examine whether rates of overdue CRC screening and no record of CRC screening are spatially clustered in Alberta. Moran’s I range from + 1 to -1, with positive values indicating spatial autocorrelation (i.e., clustering of similar rates) while negative rates indicate similar rates are located far away from each other (i.e., dispersion). A score nearing 0 indicates spatial randomness [25]. Presence of spatial autocorrelation was determined based on a *p*-value of less than 0.05.

Finally, to detect where spatial clustering is occurring, we used local Getis-Ord Gi* hot-spot analysis to determine the location and magnitude of spatial autocorrelation. Statistically significant clusters of high values are called hot spots and clusters of low values are called cold spots. Optimized Getis-Ord Gi* hot-spot analysis was used to identify areas of statistically significant clusters of LGAs with high rates of overdue or no record of CRC screening (i.e., hot spots). The optimized hot spot analysis determined the optimal fixed distance threshold and nearest neighbors as the function to conceptualized space for our hot-spot analysis. For the no record of screening outcome, the threshold distance is set at approximately 263 km, and 8 nearest neighbours. Two LGAs were excluded as outliers. Using this approach only 10.6% LGAs had less than 8 neighbours. For the overdue for screening outcome, the threshold distance is set at approximately 315 km, and 8 nearest neighbours. Two LGAs were excluded as outliers. Using this approach only 5.3% LGAs had less than 8 neighbours. Statistically significant hot and cold spots were selected using a false discovery rate (FDR) that corrects for multiple tests and spatial dependence. All spatial analysis was conducted using ArcGIS Pro 2.6.3.

## Results

### Characteristics of study population

Overall, 65% of Albertans aged 52 years or older in 2019 were CUTD on their CRC screening, 21% were overdue for CRC screening and 14% had no record of CRC screening.

Based on Table 1, all three groups differed significantly on many characteristics. Notably, those who are CUTD with screening tended to be older. Most of those with NRS were male (54.2%), while those who were CUTD (51.1%) or overdue (50.0%) for screening tended to be female. Those who were CUTD had a higher number of visits to their family doctor. Those with NRS had higher percent of individuals (36.5%) with no continuity of care and had higher percent (40.3%) of individuals who were health system non-users. Those who were CUTD had higher percent of individuals with low material (19.2%) and social (21.0%) deprivation. Geographically, those who were CUTD had higher percent of individuals living in Calgary zone (38.8%) and a low percent of people living in rural and remote regions (1.7%), while those with NRS had a higher percent of individuals living in metro regions (53.6%).

**Table 1:** Characteristics of Study Sample (n = 919,939)

	Current Up-To-Date n = 594,946 (*p* = 65%)	No Record of Screening n = 195,003 (*p* = 21%)	Overdue n = 129,990 (*p* = 14%)
**DEMOGRAPHIC FACTORS**			
**Age**			
Median (IQR)	62.00 [57.00, 67.00]	59.00 [55.00, 64.00]	61.00 [57.00, 66.00]
52–59	218,688 (36.8)	104,446 (53.6)	55,655 (42.8)
60–69	278,777 (46.9)	71,687 (36.8)	56,420 (43.4)
70+	97,481 (16.4)	18,870 (9.7)	17,915 (13.8)
**Sex**			
Male	290,721 (48.9)	105,763 (54.2)	64,957 (50.0)
Female	304,225 (51.1)	89,240 (45.8)	65,033 (50.0)
**HEALTH SYSTEM FACTORS**			
**Number visits to a Family Doctor**			
Median (IQR)	21.00 [13.00, 33.00]	12.00 [5.00, 23.00]	17.00 [9.00, 29.00]
**Continuity of Care**			
High UPC	124,982 (21.0)	27,702 (14.2)	26,522 (20.4)
Mod UPC	72,164 (12.1)	17,184 (8.8)	16,251 (12.5)
Low UPC	383,478 (64.5)	79,016 (40.5)	78,274 (60.2)
No UPC	14,322 (2.4)	71,101 (36.5)	8943 (6.9)
**Auto travel distance to closest lab in minutes**			
Between 0 and 10 min	436,165 (73.3)	145,823 (74.8)	96,019 (73.9)
10 to 20 min	119,321 (20.1)	35,415 (18.2)	24,346 (18.7)
20 to 30 min	24,530 (4.1)	7880 (4.0)	5923 (4.6)
30 min or more	14,930 (2.5)	5885 (3.0)	3702 (2.8)
**Health Profile Group**			
Health System Non-User	25,901 (4.4)	78,373 (40.2)	22,637 (17.4)
Health System User with No Health Conditions	43,837 (7.4)	12,756 (6.5)	10,566 (8.1)
Major Acute	15,484 (2.6)	3513 (1.8)	3053 (2.3)
Major Cancer	6285 (1.1)	1000 (0.5)	1080 (0.8)
Major Chronic	21,610 (3.6)	4152 (2.1)	3999 (3.1)
Major Mental Health	8141 (1.4)	2608 (1.3)	2165 (1.7)
Minor Acute	192,995 (32.4)	43,223 (22.2)	37,464 (28.8)
Minor Chronic	91,311 (15.3)	15,895 (8.2)	15,886 (12.2)
Moderate Acute	60,566 (10.2)	10,546 (5.4)	10,868 (8.4)
Moderate Chronic	90,849 (15.3)	14,334 (7.4)	15,068 (11.6)
Obstetrics	134 (0.0)	< 50 (0.0)	< 50 (0.0)
Other Cancer	10,199 (1.7)	1173 (0.6)	1361 (1.0)
Other Mental Health	27,515 (4.6)	7334 (3.8)	5767 (4.4)
Palliative	98 (0.0)	< 50 (0.0)	< 50 (0.0)
**SOCIOECONOMIC FACTORS**			
**Pampalon Material Deprivation**			
Missing	24,606 (4.1)	9183 (4.7)	5806 (4.5)
1 – Least Deprived	114,165 (19.2)	33,831 (17.3)	21,411 (16.5)
2	109,040 (18.3)	31,322 (16.1)	22,205 (17.1)
3	112,694 (18.9)	35,412 (18.2)	24,481 (18.8)
4	123,442 (20.7)	40,816 (20.9)	28,274 (21.8)
5 – Most Deprived	110,999 (18.7)	44,439 (22.8)	27,813 (21.4)
**Pampalon Social Deprivation**			
Missing	24,606 (4.1)	9183 (4.7)	5806 (4.5)
1 – Least Deprived	124,683 (21.0)	32,465 (16.6)	23,315 (17.9)
2	94,178 (15.8)	26,371 (13.5)	18,974 (14.6)
3	107,426 (18.1)	33,444 (17.2)	23,598 (18.2)
4	121,704 (20.5)	41,942 (21.5)	27,928 (21.5)
5 – Most Deprived	122,349 (20.6)	51,598 (26.5)	30,369 (23.4)
**GEOGRAPHIC FACTORS**			
**Alberta Health Zones**			
Calgary	230,983 (38.8)	73,965 (37.9)	44,062 (33.9)
Central	71,831 (12.1)	23,184 (11.9)	18,545 (14.3)
Edmonton	187,286 (31.5)	62,765 (32.2)	43,773 (33.7)
North	57,121 (9.6)	22,948 (11.8)	14,729 (11.3)
South	47,725 (8.0)	12,141 (6.2)	8881 (6.8)
**Urban/rural Continuum**			
Metro	303,756 (51.1)	104,526 (53.6)	64,688 (49.8)
Moderate Metro Influence	92,602 (15.6)	25,287 (13.0)	18,876 (14.5)
Moderate Urban Influence	14,134 (2.4)	3946 (2.0)	3279 (2.5)
Rural	96,786 (16.3)	31,239 (16.0)	23,233 (17.9)
Rural Centre Area	24,401 (4.1)	8392 (4.3)	5712 (4.4)
Rural Remote	10,362 (1.7)	4808 (2.5)	2979 (2.3)
Urban	52,905 (8.9)	16,805 (8.6)	11,223 (8.6)

### Factors associated with Colorectal cancer screening

Table 2 shows the results of the multinomial logistic regression analyses examining factors associated with having no record of or overdue for screening compared to those who are CUTD (i.e., the referent group) for screening. Those in older age groups (i.e., 60–69 years of age and 70 years of age or older) were less likely to have no record of screening compared to younger age groups. The odds of having no record of screening were over 9 times (OR: 9.30; 95% CI: 9.07 to 9.53) higher in those with no UPC compared to those with a high UPC (i.e., consistently seeing the same physician across previous appointments). Similarly, the odds of having no record of screening were 4.5 times (OR: 4.53: 95% CI: 4.40 to 4.65) higher among health system non-users compared to those who used the health system and had no health conditions. There was also a gradient in social and material deprivation, where individuals living in more deprived areas tend to have higher odds of having no record of screening, compared to those who live in less deprived areas. There was also a gradient where individuals who lived farther from a lab facility were more likely to have no record of screening. We see a similar pattern in these health system and socioeconomic factors when comparing currently up to date screening versus those overdue with screening as well, but with smaller associations. Individuals in Edmonton zone have higher odds of having no record of and overdue for CRC screening compared to those in Calgary zone. Finally, individuals in rural and remote areas have higher odds of being overdue for CRC screening compared to those in metro areas.

**Table 2:** Factors associated with no record of colorectal cancer screening and overdue for screening, fully adjusted multinomial logistic regression

	No Record of Screening (ref = Up-to-date)	Overdue (ref = Up-to-date)
	Odds Ratios (95% Confidence Interval)	
**Age**		
52–59	Ref	Ref
60–69	0.54 *** (0.53–0.54)	0.82 *** (0.80–0.83)
70+	0.44 *** (0.43–0.45)	0.78 *** (0.76–0.79)
**Sex**		
Female	Ref	Ref
Male	1.01 * (1.00–1.02)	0.99 (0.98–1.00)
**Continuity of Care**		
High UPC	Ref	Ref
Low UPC	0.97 *** (0.95–0.98)	1.01 (0.99–1.03)
Mod UPC	1.02 *** (1.00–1.04)	1.04 ** (1.01–1.06)
No UPC	9.30 *** (9.07–9.53)	1.45 *** (1.4–1.5)
**Auto travel distance to closest lab in minutes**		
Between 0 and 10 min	0.93 *** (0.92–0.95)	0.96 *** (0.94–0.98)
10 to 20 min	Ref	Ref
20 to 30 min	1.05 ** (1.02–1.09)	1.08 *** (1.04–1.12)
30 min or more	1.17 *** (1.13–1.22)	1.07 ** (1.03–1.12)
**Health Profile Group**		
Health System Non-User	4.53 *** (4.40–4.65)	3.38 *** (3.28–3.48)
Health System User with No Health Conditions	Ref	Ref
Major Acute	0.91 *** (0.87–0.95)	0.84 *** (0.80–0.88)
Major Cancer	0.69 *** (0.64–0.74)	0.75 *** (0.70–0.81)
Major Chronic	0.82 *** (0.79–0.85)	0.80 *** (0.76–0.83)
Major Mental Health	1.14 *** (1.08–1.20)	1.07 * (1.02–1.13)
Minor Acute	0.81 *** (0.79–0.83)	0.81 *** (0.79–0.83)
Minor Chronic	0.70 *** (0.68–0.72)	0.74 *** (0.72–0.76)
Moderate Acute	0.71 *** (0.69–0.73)	0.75 (0.73–0.78)
Moderate Chronic	0.69 *** (0.67–0.71)	0.72 *** (0.70–0.74)
Obstetrics	0.35 *** (0.19–0.64)	0.60 * (0.37–0.98)
Other Cancer	0.53 *** (0.49–0.56)	0.61 *** (0.58–0.65)
Other Mental Health	0.98 (0.94–1.01)	0.86 *** (0.83–0.89)
Palliative	1.45 (0.95–2.22)	1.25 (0.81–1.93)
**Pampalon Social Deprivation**		
1 – Least Deprived	Ref	Ref
2	1.04 *** (1.02–1.07)	1.04 *** (1.02–1.06)
3	1.17 *** (1.15–1.20)	1.11 *** (1.09–1.14)
4	1.27 *** (1.24–1.29)	1.16 *** (1.14–1.18)
5 – Most Deprived	1.44 *** (1.42–1.47)	1.23 *** (1.21–1.26)
**Pampalon Material Deprivation**		
1 – Least Deprived	Ref	Ref
2	1.08 *** (1.06–1.10)	1.10 *** (1.08–1.12)
3	1.15 *** (1.13–1.18)	1.15 *** (1.12–1.17)
4	1.22 *** (1.19–1.24)	1.19 *** (1.17–1.22)
5 – Most Deprived	1.50 *** (1.47–1.53)	1.31 *** (1.29–1.34)
**Alberta Health Zones**		
Calgary	Ref	Ref
Central	0.99 (0.95–1.02)	1.26 *** (1.21–1.31)
Edmonton	1.12*** (1.11–1.14)	1.24 *** (1.22–1.26)
North	1.01 (0.97–1.05)	1.18 *** (1.13–1.23)
South	0.82 *** (0.79–0.86)	0.94 * (0.90–0.99)
**Urban/rural Regions**		
Metro	Ref	Ref
Moderate Metro Influence	0.89 *** (0.87–0.90)	0.95 *** (0.94–0.97)
Moderate Urban Influence	0.88 *** (0.83–0.93)	1.01 (0.95–1.07)
Rural	0.88 *** (0.85–0.91)	0.98 (0.94–1.01)
Rural Centre Area	1.02 (0.97–1.06)	0.99 (0.94–1.04)
Rural Remote	1.04 (0.98–1.11)	1.12 *** (1.05–1.19)
Urban	0.97 (0.93–1.02)	0.97 (0.92–1.01)

* *p* < 0.05, ** *p* < 0.001, ***p < 0.0001

### Findings from spatial analyses

**Fig. 2:**
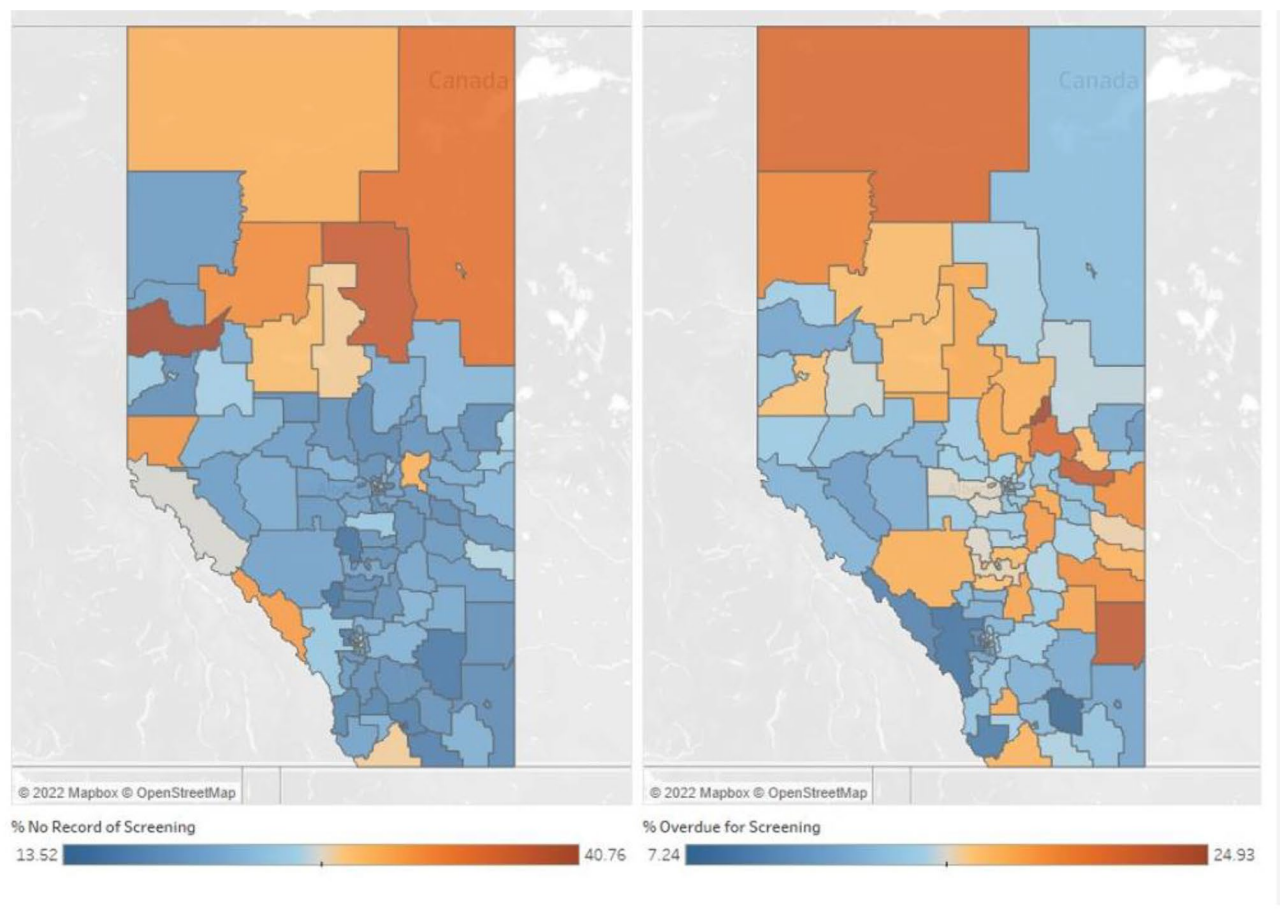
Percent of individuals with no record of (left) and overdue for (right) colorectal cancer screening by Local Geographic Area in Alberta

Results (see Appendix A) from the Moran’s I index indicate that the distribution of individuals with no record of CRC screening and those overdue for CRC screening are statistically significantly spatially autocorrelated (i.e., 99% probability that the distribution of individuals with no record of and overdue for CRC screening forms a clustered pattern by LGA in Alberta, Canada). There was a wide variation in the percent of individuals with no record of CRC screening across each LGAs, ranging from 13.52% in Lethbridge- West (Southeastern Alberta) to 40.76% in Spirit River (Northwestern Alberta) with most of the LGAs higher percent of no record of screening concentrated in the North and Central zones (Fig. 2). Figure 2 also presents the percent of individuals who are overdue for CRC screening across each LGAs which ranges from 7.24% in Taber MD (Southeastern Alberta) to 24.93% in Boyle (Northeastern Alberta). Each LGA was classified based on rural-urban regions.

**Fig. 3:**
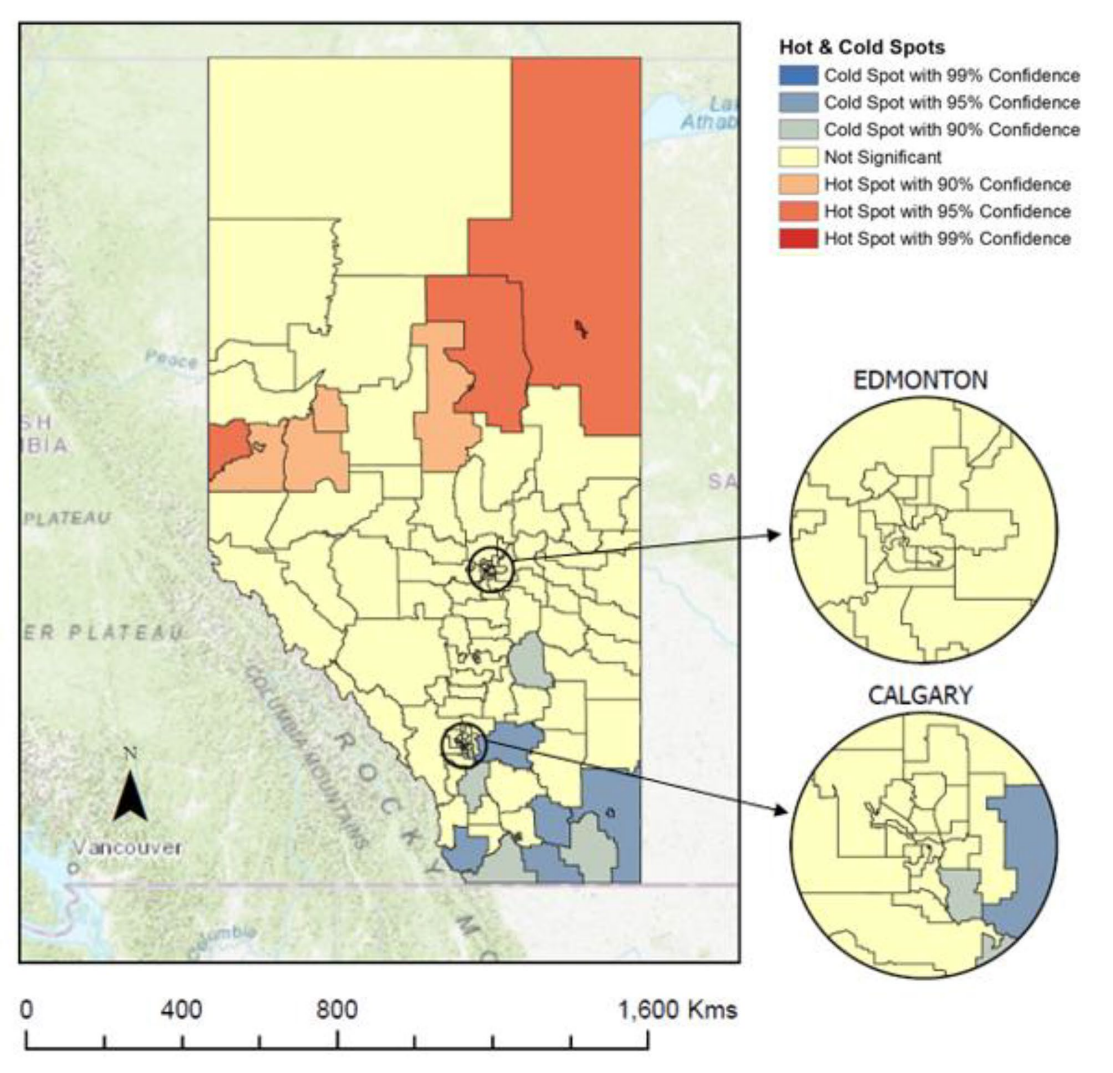
Hot and cold spots for individuals who have no record of colorectal cancer screening by Local Geographical Areas in Alberta, Canada

For individuals with no record of CRC screening, there were 23 statistically significant LGAs identified as hot (in red) and cold (in blue) spots. Results from Fig. 3 indicate that there are statistically significant hot spots (i.e., high rates of individuals with no record of CRC screening are surrounded by LGAs also with high rates of individuals with no record of CRC screening) of individuals who have no record of CRC screening grouped in the North zone. There are three statistically significant cold spots (i.e., low rates of individuals with no record of CRC screening are surrounded by LGAs also with low rates of individuals with no record of CRC screening) of individuals who have no record of CRC screening, one in the Central zone, one in the Calgary zone and one in the South zone.

**Fig. 4:**
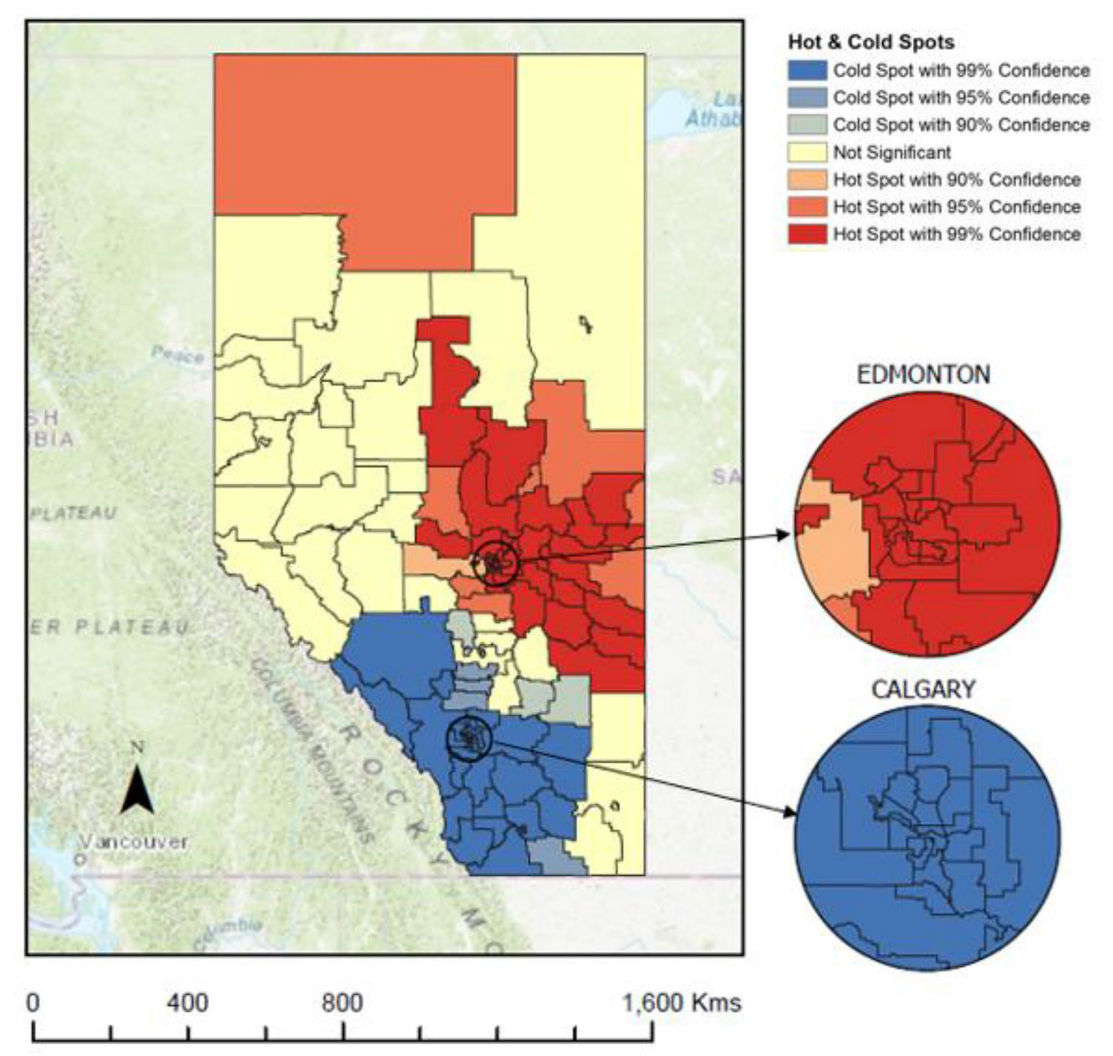
Hot and cold spots for individuals who are overdue for colorectal cancer screening by Local Geographical Area in Alberta, Canada

There were 102 statistically significant LGAs with individuals overdue for CRC screening identified as hot (in red) and cold (in blue) spots (Fig. 4). There was a large statistically significant hot spot of individuals who are overdue for CRC screening that crosses across three geographic zones (North, Central, and Edmonton). There was also a large statistically significant cold spots that crosses three zones (Central, Calgary and South).

## Discussion

Overall, our study found that approximately 65% of Albertans were CUTD with their CRC screening. This finding aligns with a recent study conducted with the Canadian Community Health survey data from Alberta that found 62.6% of Albertans were CUTD on their CRC screening [7]. In terms of sociodemographic factors, older aged individuals (60+) were less likely to have no record of or be overdue for screening compared to those aged 52–59. This finding may indicate a need for increased health marketing on the benefits and process of CRC screening targeted at people at or entering the lower decade of the age limit for CRC screening (i.e., people approaching their 50s). Previous studies have observed lower levels of knowledge about CRC screening among younger individuals [26] and addressing this knowledge gap is crucial as recent trends have indicated increased incidence of CRC among younger populations (< 50 years of age) [27] and higher prevalence of late-stage diagnosis among younger populations [28]. We found that health profile group was an important factor associated with CRC screening, which suggests that certain health profiles may face distinct barriers to screening such as competing health priorities. We also observed an increasing trend, such that individuals living in more socially and materially deprived areas were more likely to have no record of or be overdue for screening even after adjustment for healthcare factors and geographic factors. This relationship has been observed in studies examining the relationship between CRC screening and deprivation in France [29], England [30] and in Ontario, Canada [31] as well as a study examining deprivation and CRC cancer prevalence in Canada [28]. Therefore, it appears that a gradient in CRC screening participation is present even in countries with healthcare systems that cover the costs associated with CRC screening.

When comparing results from the multinomial logistic regression and the geospatial analysis, we found that the North zone had clusters of LGAs with high prevalence of no record of screening, yet the results from the adjusted multinominal logistic regression indicated that ORs of having no record of screening were not statistically significant in the North zone. Therefore, while the geospatial analysis gives us insights into the areas to target for additional interventions to increase CRC screening uptake, those interventions should aim to address healthcare and sociodemographic barriers that are driving CRC screening inequalities in those areas. For example, our study found that those that did not have a usual provider of care and those identified as healthcare non-users were much more likely to have no record of and overdue for screening. These results mirror a similar study using the Canadian Community Health Survey, which found that respondents who reported having a regular health care provider was associated with being up to date for CRC screening [7]. Moreover, within the Alberta healthcare context, continuity is pivotal, emphasizing the need for seamless patient-provider relationship. Consistent with this, our data indicates that heightened continuity (i.e., seeing the same provider at each visit) can positively influence CRC screening adherence, which may be attributed to enhanced patient-provider communication, regular screening reminders, and increased trust in providers advice [32]. Availability of family doctors per resident continue to be a concern in Canada [33]. To target those without a regular provider of care and low continuity of care, public health interventions such as mobile screening clinics may be an innovative solution to increase CRC screening in Alberta. Mobile screening clinics that screen for multiple cancers in one appointment have been shown to be successful in increasing screening rates among women in a pilot conducted in rural and remote regions in Alberta [34].

We found that those who lived 30 min or more in driving distance from the closest laboratory services were more likely to have no record of and overdue for screening. Our results mirror those to a recent study in Norway, which found that driving time to a screening center was a significant predictor of colorectal cancer screening participation [35]. However, several studies in the USA did not find that distance to the screening clinic was a significant factor. For example, one study in Dallas County, Texas, examined the role of distance to the screening clinic on screening status but did not find it to be significant factor [9]. This non-significant finding may be due to the study’s sample living primarily in an urban area. Another study of CRC screening status conducted in Missouri, found that geographic variations across the state were partially but not completely explained by area-level poverty rates [36]. In our study, we observed a trend between increased auto travel distance and having no record of screening. However, it is important to note that over 90% of our sample were within 30 min of driving time to a laboratory service. Thus, our results and those found in the literature may suggest that distance (or driving time) to the laboratory service to receive a FIT kit and then the return trip to drop off completed FIT kit plays a unique role on CRC screening dependent on the jurisdiction under-study and additional research is needed on this factor. To overcome this distance barrier, public health interventions could focus on rolling out (or expanding) mail-in FIT kits in areas with low CRC screening to reduce the need to drive to complete CRC screening. In Alberta, mail-in FIT kits has been implemented however a fulsome evaluation is needed to understand the impact of this program on geographical inequities in CRC screening. Mail-in FIT kits have been shown to be feasible as CRC screening [37] and may be able to be address the barrier around returning the FIT kit. Increasing FIT kit distribution in primary care clinics may be able to address the barrier around receiving the FIT kit but additional research is needed to determine how to engage individuals with low attachment to the health system. Therefore, to address these CRC screening barriers a multicomponent approach may be warranted, results for the geospatial analysis can provide insights into specific regions to target, tailor and implement a novel multicomponent intervention. For future research, a deeper exploration into the reasons for differences in CRC screening is crucial. Qualitative studies could provide valuable insights into the barriers and facilitators for CRC screening, especially among younger or more vulnerable cohorts (i.e., those with less access to family care providers or located in rural and remote regions). Understanding their perceptions, knowledge gaps, and motivations can guide more effective and targeted interventions.

### Strengths

The present study has several strengths. First, this study was conducted using a large health administrative database, which covered the entire province of Alberta. This strength therefore removes any recall bias associated with survey-based studies. Therefore, our study builds on existing literature in Alberta that examined healthcare factors and CRC screening using health record data rather than self-reported data and contributes to the literature by examining additional sociodemographic and geographic factors using administrative data. Second, this is the first paper to examine CRC screening in Canada using geospatial methods, which allows public health researchers to use this information to prioritize regions for intervention. Moreover, analysis of sociodemographic and health system factors that influence CRC screening participation provides additional information on the contextual barriers experienced in those regions with lower rates of CUTD CRC screening. Third, to the best of our knowledge, this is the first study that examined the relationship between total driving distance (as opposed to straight-line (Euclidean) distance) to laboratory services and CRC screening status, which reflects a more realistic picture of the time taken by patients to complete CRC screening. The laboratory is an important component of the CRC screening pathway in Alberta as the individual must receive and drop off their FIT sample at laboratory services to complete their screening.

### Limitations

The study also has several limitations. First, the screening dataset only contained information on completed FIT and did not contain information on whether a FIT requisition was made. Future research could explore the gap between requisition and completion of FIT screening and the role of sociodemographic, geographic, or healthcare factors in explaining this gap. Moreover, we chose to include only those Albertans registered with the province for 10 consecutive years to ensure comprehensive capture of screening records and minimize misclassification of the screening status. This criterion would have led to the systematic exclusion of recent newcomers to Alberta and future studies should consider this.

Second, the CUTD screening category includes individuals who have had a colonoscopy within the last 10 years, therefore, there is the chance that the CUTD group misclassified individuals as such if they are high-risk individuals who have not had a colonoscopy every 5 years, resulting in an overestimate of the CUTD group. This is a limitation of our dataset as we were unable to identify high-risk individuals, but we do not believe this to be a large overestimation as they typically know they are high risk and are receiving regular colonoscopies [38].

Third, our multinomial logistic regression models were built using a small number of health system and sociodemographic factors and therefore there are likely unexamined factors (e.g., race/ethnicity, language, cultural factors) that could be potential confounders. Specifically, factors like race/ethnicity may introduce nuances in healthcare trust and access, while language barriers can impede effective patient-provider communication. Cultural beliefs might also shape preventive care perceptions, potentially influencing CRC screening behaviors.

Fourth, our analysis only includes GP visits and the UPC variable only includes GP visit, we do not have data on nurse practitioners’ visits which is becoming increasingly common to increase access to primary health care outside of regular business hours and in rural and remote regions [39] this might result in a under estimation in the association between healthcare access and CRC screening in some regions.

Fifth, while our study included driving distance to laboratory services as a factor influencing CRC screening, we did not examine driving distance to primary care provider which is also an important step in the CRC screening pathway. Finally, the context of our study, rooted in Alberta’s healthcare system, offers findings pertinent to settings with similar healthcare dynamics and population profiles. While our inclusion criteria, especially concerning the duration of Alberta registration, may pose generalizability constraints, the associations we observed, particularly around having a regular and consistent care provider and individual factors in relation to CRC screening, might resonate among public health systems that may be struggling with low availability of family care providers.

## Conclusions

Our study showed important variation in colorectal cancer screening participation across sociodemographic, health system and geographical characteristics and identified areas with higher proportions of individuals who have no record of screening or under-screened in Alberta, Canada. These findings can be used to provide insights into not only the geographic areas to target for additional interventions to increase CRC screening, but also the type of barriers that those interventions should aim to address in Alberta.

### Electronic supplementary material

Below is the link to the electronic supplementary material.

Supplementary Material 1

## Data Availability

The data that support the findings of this study are available from Alberta Health Services, but restrictions apply to the availability of these data, which were used under license for the current study, and so are not publicly available. Data are however available from the corresponding author upon reasonable request and with permission of Alberta Health Services.

## Abbreviations

AHS: Alberta Health Services
IQR: Interquartile Range
FDR: False Discovery Rate
FIT: Fecal Immunochemical Tests
CRC: Colorectal Cancer
UPC: Usual Provider of Care
CUTD: Currently Up-To-Date
LGA: Local Geographic Area
PHN: Personal Healthcare Number
GP: General Practitioner
ULI: Unique Lifetime Identifier
ACRCSP: Alberta Colorectal Cancer Screening Program
NACRS: National Ambulatory Care Reporting System
CIHI: Canadian Institutes of Health Information
DAD: Discharge Abstract Database
ACCS: Ambulatory Care Classification System
DA: Dissemination Area
AHCIP: Alberta Health Care Insurance Plan
DIMR: Data, Integration, Measurement & Reporting
