# Supplementary material for: Geospatial analysis and participant characteristics associated with colorectal cancer screening participation in Alberta, Canada: a population-based cross-sectional study
Source: BMC Health Serv Res. 2023 Dec 21;23:1454. doi: 10.1186/s12913-023-10486-8 (PMC10740253; doi:10.1186/s12913-023-10486-8)
Supplement: Supplementary file 1 — Supplementary Material 1 [file 12913_2023_10486_MOESM1_ESM.docx]

**Appendix A**: Results of Moran’s I Index of Spatial Autocorrelation

|  | **No Record of Colorectal Cancer Screening** | **Overdue for Colorectal Cancer Screening** |
| --- | --- | --- |
| Moran’s I Index | 0.215 | 0.190 |
| Z-Score | 7.860 | 6.957 |
| P-value | < 0.0001 | < 0.0001 |
